# Access to abortion under the heath exception: a comparative analysis in three countries

**DOI:** 10.1186/s12978-018-0548-x

**Published:** 2018-06-13

**Authors:** Stephanie A. Küng, Blair G. Darney, Biani Saavedra-Avendaño, Patricia A. Lohr, Laura Gil

**Affiliations:** 1 Mailman School of Public Health, Heilbrunn Department of Population and Family Health, Columbia University, New York, NY USA; 2 Oregon Health & Science University Department of Obstetrics and Gynecology, 3181 SW Sam Jackson Park Rd, Portland, OR 97239 USA; 3 Instituto Nacional de Salud Pública, Centro de Investigacion en Sistemas de Salud, Cuernavaca, Mexico; 4 British Pregnancy Advisory Service, Stratford Upon Avon, UK; 5 Fundación Oriéntame/ESAR, Bogotá, Colombia

**Keywords:** Abortion, Mexico; Colombia, Great Britain, Health exception, Law and policy, Public health, Family planning, Causal Salud

## Abstract

**Background:**

Despite Britain, Colombia, and some Mexican states sharing a health exception within their abortion laws, access to abortion under the health exception varies widely. This study examines factors that result in heterogeneous application of similar health exception laws and consequences for access to legal abortion. Our research adds to previous literature by comparing implementation of similar abortion laws across countries to identify strategies for full implementation of the health exception.

**Methods:**

We conducted a cross-country comparative descriptive study synthesizing data from document and literature review, official abortion statistics, and interviews with key informants. We gathered information on the use and interpretation of the health exception in the three countries from peer-reviewed literature, court documents, and grey literature. We next extracted public and private abortion statistics to understand the application of the law in each setting. We used a matrix to synthesize information and identify key factors in the use of the law. We conducted in-depth interviews with doctors and experts familiar with the health exception laws in each country and analyzed the qualitative data based on the previously identified factors.

**Results:**

The health exception is used broadly in Britain, somewhat in Colombia, and very rarely in Mexican states. We identified five factors as particularly salient to application of the health exception in each setting: 1) comprehensiveness of the law including explicit mention of mental health, 2) a strong public health sector that funds abortion, 3) knowledge of and attitudes toward the health exception law, including guidelines for physicians in providing abortion, 4) dissemination of information about the health exception law, and 5) a history of court cases that protect women and clarify the health exception law.

**Conclusions:**

The health exception is a valuable tool for expanding access to legal abortion. Differences in the use of the health exception as an indication for legal abortion result in wide access for women in Britain to nearly no access in Mexican states. Our findings highlight the difference between theoretical and real access to legal abortion. The interpretation and application of the health exception law are pivotal to expanding real access to abortion.

## Resumen

**Propósito:**

A pesar de que Gran Bretaña, Colombia y algunos estados mexicanos comparten una causal salud dentro de sus leyes de aborto, el acceso al aborto bajo la causal varía ampliamente. Este estudio examina los factores que resultan en la aplicación heterogénea de leyes similares y las consecuencias para el acceso al aborto legal. Nuestra investigación se suma a la literatura anterior comparando la implementación de leyes entre países para dilucidar estrategias para la implementación total.

**Métodos:**

Realizamos un estudio descriptivo comparativo entre países que sintetizó datos de la revisión de documentos, estadísticas oficiales, y entrevistas con informantes claves. Recopilamos información sobre el uso y la interpretación de la causal salud en los tres países haciendo referencia a literatura revisada por pares, documentos judiciales, y literatura gris. Extraemos estadísticas públicos y privados para comprender la aplicación de la ley en cada entorno. Usamos una matriz para sintetizar información e identificar factores clave en el uso de la causal salud. Realizamos entrevistas en profundidad con médicos y expertos familiarizados con las leyes y analizamos los datos cualitativos en función de los factores previamente identificados.

**Resultados:**

La causal salud se usa ampliamente en Gran Bretaña, a veces en Colombia, y casi nunca en los estados mexicanos. Identificamos cinco factores relevantes para la aplicación de la causal salud: 1) la exhaustividad de la ley, incluyendo la mención explícita de la salud mental, 2) un fuerte sector de salud pública que financia el aborto, 3) conocimiento y actitudes hacia la ley, incluyendo directrices para los médicos, 4) difusión de información sobre la causal salud, y 5) un historial de casos judiciales que protegen a las mujeres y clarifican la ley.

**Conclusiones:**

La causal salud es una herramienta valiosa para ampliar el acceso al aborto legal. Las diferencias en el uso de la causal dan como resultado un amplio acceso para mujeres en Gran Bretaña a casi ningún acceso en los estados mexicanos. Nuestros hallazgos resaltan la diferencia entre el acceso teórico y real al aborto legal. La interpretación y la aplicación de la causal salud son cruciales para expandir el acceso real al aborto.

## Plain English summary

In Great Britain (England, Scotland, and Wales), Colombia, and some Mexican states, abortion is legal when the pregnancy poses a risk to the woman’s health. However, the application of this health exception varies widely among the three countries. This study identifies factors that result in such different application of similar health exception laws. We also discuss the consequences of different application of the health exception for access to legal abortion.

We reviewed literature, court documents, and official abortion statistics, and conducted in-depth interviews with doctors and other experts familiar with the health exception laws in each country. We synthesized this information and identified key factors to the use and non-use of the health exception.

We found that the health exception is used broadly in Britain to provide access to abortion services, somewhat in Colombia, and very rarely in Mexican states. We identified five factors that influence how the health exception is applied in each country setting: 1) the definition of health, with explicit mention of mental as well as physical health, 2) a strong public health sector that provides abortion services, 3) knowledge of the health exception law, including clear guidelines for physicians, 4) dissemination of information about the health exception law, and 5) a history of court cases that protect women and clarify the health exception law.

Differences in the use of the health exception to provide access to legal abortion result in wide access to abortion services for women in Britain to nearly no access to abortion in Mexican states. The interpretation and application of the health exception law are important to expanding access to abortion.

## Background

Unsafe abortion has been recognized as a global public health issue for over two decades [1]. Every year in developing countries, 5 million women are hospitalized due to complications from unsafe abortion, and in Latin America and the Caribbean at least 10% of maternal deaths in the region are attributed to unsafe abortion [2]. This is a huge strain on health systems, not to mention the financial, physical, and mental costs to women and their families [3–5].

In many countries where access to abortion is restricted, abortion laws delineate exceptional circumstances under which abortion can be performed without risk of prosecution. The most common exceptions are risk to the woman’s life, fetal anomaly, risk to the woman’s health, rape, and incest [6]. While abortion provided to save the life of the woman is legal in most countries in the world, the health exception, defined here as preserving the physical *and/or* mental health of the woman, is law in 36% of countries [6].

The gap between abortion laws and their application has been documented, but has not been extended to cross-country application of the health exception [7]. Colombia, Great Britain, and some states in Mexico allow abortion when the physical and/or mental health of the woman is at risk [6]. Britain’s law (the Abortion Act of 1967), allows abortion to 24 weeks’ gestation when two doctors agree that the risk to the mental or physical health of the woman or her existing children is greater with continuance of a pregnancy compared to that associated with termination [8]. Colombia’s abortion law was amended in 2006 to legalize abortion at the federal level under three conditions, including rape, fetal anomaly, and when the woman’s health is at risk; this was the first time a court reviewed the constitutionality of abortion under a human rights framework and concluded it was unconstitutional [9]. Abortion in Mexico is not governed under federal law; each of Mexico’s 32 states has its own abortion laws, and therefore the exceptions vary by state. In March 2016, new federal guidelines that do not require police or clinical verification of rape under the rape exception were passed, although this federal guideline is in direct conflict with some state laws [10]. Of Mexico’s 32 states, 14 allow abortion when the woman’s health is at risk, and there is no federal guidance for the health exception [11]. Mexico City is the only state where abortion is legal on demand, and only in the first trimester [12]. The purpose of this study was to identify factors that contribute to heterogeneous application of similar health exception laws in Great Britain, Colombia, and Mexico.

## Methods

We conducted a cross-country comparative descriptive study synthesizing data from document and literature review, official abortion statistics, and interviews with key informants. We chose three countries where access to abortion under the health exception ranges from very limited (Mexico), somewhat limited (Colombia), and very expansive (Great Britain), despite the laws as written being similar. Experts on our team also identified that these three countries would be ideal for comparison due to differences in the historical contexts out of which these laws were passed, such as religious environment and the scope of the laws (federal vs. state). We reviewed literature, including peer-reviewed articles, government documents, court jurisprudence, and grey literature pertaining to the health exception in the three countries with the aim of identifying and abstracting available data about access to and use of the health exception in each setting. We documented relevant jurisprudence around abortion to identify protection afforded to doctors and women utilizing the health exception in the courts. We identified factors, practices, or events that were similar or different across countries and developed a matrix to display synthesized data and permit cross-country comparisons.

We next extracted data from official statistics or private sector statistics where available (Colombia). We understand that abortion statistics in contexts where reporting is poor undercount abortion incidence, but they do reflect the official reporting of abortion service provision. In Mexico we utilized data from the Government Statistics Office (INEGI), which collects data on hospitalizations at public hospitals [13]. In Colombia we analyzed administrative data from the Sistema de Información de Prestaciones de Salud (RIPS) [14], as well as data collected by a private abortion provider in Bogotá. In Britain we analyzed data published annually by the Departments of Health of England & Wales, and Scotland [15, 16].

Our third phase relied on qualitative data from key informant interviews. We used a convenience sample of providers from Mexico, Colombia, and Britain who have experience employing the health exception, academic scholars who have studied the health exception, and NGO partners who focus on expanding access to abortion under the health exception. The aim of this third phase was to supplement and verify findings from the document review and quantitative data. We developed the interview guide based on findings from the document review, and focused on two key thematic areas: knowledge of the health exception in their state/country, and barriers to use of the health exception. We conducted 17 interviews in Spanish or English (Mexico [*n* = 6], Britain [*n* = 7], and Colombia [*n* = 4]). The majority (82%) of in-depth interviews were held with doctors, the rest were with academic scholars or NGO partners. Interviews were recorded and transcribed. We developed a codebook using a priori themes informed by the document review and also allowed for emergent themes. The first author developed the first iteration of the codebook; it was subsequently revised and collapsed by consensus with the second and third authors, and validated by the fourth and senior authors. We used Dedoose version 7.5.9, an online coding platform, to organize the qualitative analysis. All participants consented to participation and the study was approved by the Comité de Ética (IRB) of the Instituto Nacional de Salud Pública in Cuernavaca, Mexico.

## Results

Table 1 summarizes general characteristics of each law, including language and specific characteristics such as dissemination of the law, access and barriers, and jurisprudence by country. Because no federal abortion law exists in Mexico, Table 1 compares the health exception laws in the 14 Mexican states in which abortion is legal under health circumstances. The health exception law and its use are generally consistent across Mexican states; differences are noted. The ‘plus’ signs indicate widespread distribution of the law and information about the law, while ‘minus’ signs indicate poor or non-existent distribution, and inclusion of both signs indicate neutrality.

**Table 1:** Characteristics of the health exception within the abortion laws of Mexico, Britain, and Colombia

Characteristic	Country		
	Britain	Colombia	Mexico
Health exception law coverage / year	Federal (applies to England, Scotland, Wales) / 1967 [34]	Federal / 2006 [35]	State / 1990–2016 [36]
Legislative precedence	Act of parliament (law reform)	Total ban declared unconstitutional under a human rights framework	Penal code reform
Language of the law	Risk, greater than if the pregnancy were terminated, of injury to the physical or mental health of the pregnant woman [34]	Risk to the life or health of the woman [22]	Severe risk to the health of the woman Risk of death or severe injury to the health of the woman [37]
Gestational age limit for the health exception	24 weeks [34]	None [35]	None [37]
Number of doctors required for approval of health exception	Two [38]	One [35]	Two [11]
Consideration of economic or social factors explicitly mentioned or allowed	Yes [38]	No	In States of Michoacán and Yucatán [39] [47]
Further barriers exist for minors securing an abortion under the health exception	No	No	Yes [40]
Mental health is explicitly mentioned in the health exception law	Yes [34]	No, but stated by the Constitutional Court [9]	No
There was widespread dissemination around the law and health exception to health care providers (including provider trainings and other education)	++ [41]	+ [42]	–
There was widespread dissemination of the health exception law to the public	++ [43, 44]	+/− [22]	–
Evidence suggests that access is as easy in the public as in the private sector	No	No	No
There are documented cases protecting women or doctors accessing or performing abortions under the health exception	Yes [34]	Yes [9]	Yes [11]
There are documented cases of women *not* being able to access abortion under the health exception	No	Yes [9]	Yes [11]
There are documented cases of women accessing abortion using the health exception law	Yes [15]	Yes [45]	Yes [46]

Triangulating our data sources (document review, statistics, and interviews), we first present a summary of the use of the health exception in each country. We identified five key factors as particularly salient to application of the health exception in each country, which we discuss in turn: 1) comprehensiveness of the law including explicit mention of mental health, 2) a strong public health sector that funds abortion, 3) knowledge of and attitudes toward the health exception among providers, including guidelines for physicians in the provision of abortion, 4) dissemination of information about the health exception law, and 5) a history of court cases that protect women and clarify the health exception law.

### Use of the health exception

The health exception is used widely in Britain, rarely in Colombia, and almost never in Mexican states. In England and Wales in 2015, 98% of all abortions were performed under the health exception, almost exclusively (99.9%) due to mental health risk [15]. In Colombia, 99.3% of abortions reported by the Department of Health in Bogotá in 2015 were performed under the health and/or life exception. However, a 2011 study estimated that despite the law, more than 400,000 illegal abortions occur each year in Colombia [5]. When comparing the number of officially reported abortions with all expected abortions, only 6% of abortions performed in Bogotá occur within the law. In Mexico, only 2483 therapeutic abortions (recommended by a doctor) were performed in the 14 states with the health exception over a nine-year period [13]. When compared to the number of estimated total abortions [17], legal abortion under exceptions accounts for a negligible 0.05% of all estimated abortions.

#### Comprehensiveness of the health exception law

Document synthesis and qualitative data revealed important distinctions in the language and subsequent interpretation of the health exception law in the three countries. The language of the British law requires doctors to weigh the mental and/or physical risks of pregnancy and delivery against that of termination to determine if a request for an abortion can be authorized. Britain’s law also explicitly allows for consideration of a woman’s “actual or foreseeable environment” when considering those risks and so could reasonably include socio-economic considerations [8]. The ambiguity of the language and determination of comparative risk was viewed by respondents as one of the strengths of the British law in that it can be and is broadly interpreted. As one respondent said,

“[the law] basically makes a balance between the risk of carrying on the pregnancy and the risk of having a termination and so the doctor has to say that it will be safer or better for the woman to have an abortion than to continue the pregnancy…the fact is actually that statistically it is safer to have an abortion than to take pregnancy to term” [*Provider, Britain*].

In contrast, mental health is not explicitly mentioned in the Colombian or Mexican laws, although both countries theoretically subscribe to the WHO definition of health as encompassing mental and social wellbeing [18]. Barring two Mexican states, neither Colombia nor Mexico’s laws explicitly allow for the consideration of economic or social circumstances under the health exception, and some Mexican states include the qualifier “severe” health risk.

Restrictive interpretations that limit the use of the health exception were addressed often by key informants in Mexico and Colombia. As one respondent in Colombia noted,

“The barrier that we face is that in many places the woman cites the health exception and the response she gets from the service providers is: but you are not sick or you are not dying…In other words, a totally restrictive interpretation of the health exception” [*NGO Partner, Colombia*].

In Mexico, one respondent echoed this restrictive interpretation:

“If the patient requests [an abortion] because there’s a malformation diagnosed by a doctor that affects the life of the mother or you know the baby doesn’t have much chance, these patients are the ones that can say ‘you know what, I don’t want to continue my pregnancy,’ but a patient with a healthy pregnancy that says ‘hey, I want an abortion,’ well no” [*Provider, Mexico*].

Health exception laws in the three settings also differ in identifying *who* is responsible for interpreting the law. Britain assigns this responsibility to doctors, identifying abortion as a medically authorized access to a service. Physicians as gatekeepers to abortion could be quite limiting, but because abortion is an explicitly medically authorized service within a health system with a broad interpretation of health and well-being, with providers who have clear guidance about the health exception law, this limitation does not manifest as a barrier in the British setting [19]. Respondents in Colombia described doctors as having the responsibility to define risk, but clarified that the right to make a decision based on that risk assessment lies with the woman. Crucial to proper functioning of the health exception in this context, then, is knowledge of the health exception among both providers and women. In Mexican states, key informants agreed that risk assessment is reserved for doctors and, in some cases, hospital ethics committees. Furthermore, Mexican states do not benefit from liberal interpretation and widespread provider knowledge of the health exception law, which limits access to abortion.

#### Strong public sector funding of abortion

Healthcare in Britain, including abortion, is funded by the National Health Service (NHS), whether delivered from an NHS hospital or an independent sector clinic working under contract to the NHS [15]. Mexico’s public health system is made up of various institutions, including the Ministry of Health (Secretaria de Salud or SSA; decentralized at the state level), and insurance for formal sector employees (IMSS) and state workers (ISSSTE) [20]. IMSS and ISSSTE are federal entities and have not affirmed their responsibility to cover abortion under the health exception (R. Schiavon, personal communication, 08/18/16). Colombia’s health system, which is made up of public, private, for profit, not for profit, and religiously affiliated insurers [21], is legally required to cover abortions that fall under the three exceptions or circumstances outlined in the 2006 law. However, our review of the literature indicates that illegal refusals to cover abortion services remain commonplace [22].

Key informants from the three settings discussed barriers unique to abortion services in the public sector. Respondents in Colombia and Mexico spoke about a dearth of resources, including staff, as well as instances of disrespectful care and providers who cite conscientious objection. Respondents in Britain discussed hospital-based providers losing their clinical skills in abortion due to abortion services occurring overwhelmingly in independent sector clinics. However, whereas in Colombia and Mexican states (with the exception of Mexico City), public sector barriers translate into extremely restricted access for women who rely on these services, the same is not true in Britain where abortion is integrated into public sector services with a broad interpretation of health and well-being and thus widely accessible.

#### Knowledge of and attitudes toward the law

Our literature review revealed very limited evidence that permit cross-country comparison on provider and public knowledge in the three countries [23–25]. However, qualitative interviews elucidated important distinctions in provider knowledge across settings. In Colombia and Mexico, all respondents commented on the lack of knowledge among providers about the health exception and its application. For example:

“…[knowledge] is very low, unfortunately it is an issue that doctors make a lot of noise about and they have not gotten to see the legal question, and very few doctors know the situation regarding the laws and the reforms that have occurred in the country” [*Provider, Mexico*].

In Britain, the political and moral conditions surrounding abortion were seen to encourage providers to know more about the law, rather than deter their interest.

“…because the health care providers feel like they’re operating under the spotlight of potential media scrutiny and so I think, I think there is an awareness that they need to be very very careful to abide by the law…they need to understand what the law says” [*Academic Scholar, Britain*].

On the topic of knowledge of the health exception law among women who are the potential beneficiaries of legal abortion services, respondents from all countries described limited knowledge. For women in Mexico and Colombia, this was concentrated in rural settings. As one key informant described,

“…many say to me: ‘no, I did not know I could do this [get a legal abortion], I didn’t know it existed.’ The lack of information is very high above all among our lower strata with less access to education and less access to information, and likewise, among the personnel or women who live isolated in rural areas” [*Provider, Colombia*].

It is important to understand these responses in the context of pervasive stigma and weak national ownership over abortion in Mexico and Colombia, which was also mentioned by respondents as impacting willingness to provide abortion, and knowledge of abortion law among women and providers. In Britain, respondents similarly commented that women had limited knowledge of the health exception underpinning the law. Notably, instead of believing abortion to be illegal, respondents perceived that women wrongly assume abortion to be legal on demand, demonstrating the comprehensiveness with which Britain has interpreted the health exception.

“So my students for example, when I teach abortion, all are almost without exception very shocked to find out how the real law looks on paper, the written law, because all they know is that if they need to access abortion services they are going to be able to do that” [*Academic Scholar, Britain*].

#### Dissemination of information about the law

Dissemination of information about abortion law is important for providers to know the laws under which they operate and for women to know and exercise their rights. In Britain, there has been widespread dissemination of the health exception law to providers, including in medical school curricula, provider trainings, and through national guidelines [8]. Information about legal access to abortion through the health exception is also widely available to the public via the NHS website and through the websites of independent sector clinics that provide abortion, and young people’s, family planning, and advocacy organizations. Key informants largely echoed the view that poor dissemination of information is not a barrier to abortion access under the health exception in Britain. Only one respondent acknowledged minor difficulties in getting information to specific communities:

“I think there is always going to be hard to reach communities and maybe the women in some ethnic communities, recent immigrants, women who don’t have good English, there are probably problems of information” [*Academic Scholar, Britain*].

The extensive coverage of the 2006 liberalization of abortion law in Colombia resulted in widespread dissemination [22]. Guidelines on the provision of abortion were drafted in consultation with feminist groups in the country, and numerous court cases have clarified the use of the health exception for providers [9]. However, respondents in Colombia overwhelmingly described disparities in access to information between urban and rural settings.

Respondents in Colombia also discussed the use of misinformation campaigns by anti-choice groups and the withholding of information by providers opposed to abortion as hampering access to accurate information. According to one respondent,

“Anti-choice groups impede, not only non-governmental organizations that carry out, let’s say, actions to oppose the right to decide, but also, especially, state actors that hold important public positions and that have taken on the task in Colombia of carrying out a campaign of misinformation and confusion, as much for providers as for women” [*NGO Partner, Colombia*].

In Mexico, scarce information exists about the legality of the health exception to abortion in the 14 states. Most information regarding abortion in Mexico focuses on Mexico City, where first trimester abortion is available on request since 2007. Both the document review and key informant interviews identify the Mexico City law as having adverse impacts on access to abortion under the health exception in other states. Following the decriminalization of abortion in Mexico City, 16 State constitutions were amended to protect life from conception, and women attempting to obtain abortions in other states are often encouraged to travel to Mexico City [26]. As one key informant described,

“I think it [the Mexico City law] is such a strong accomplishment that it obscures the health exception…such that there is not even interest in broadcasting it, the health care providers don’t broadcast it and in the case of the interior of the country, above all the organizations are broadcasting the Mexico City law more, so that women can have access to legal abortion on demand” [*NGO Partner, Mexico*].

Guidelines for providers regarding abortion provision under the health exception in states outside of Mexico City are similarly lacking, with many informants expressing a lack of clarity about which guidelines are applicable or whether guidelines exist. Stigma was explicitly mentioned as a barrier to information, as talking about abortion is considered taboo.

#### Jurisprudence that clarifies the law

We analyzed court cases to assess if and how doctors and women alike have been protected under the health exception law in Britain, Colombia, and Mexico. The landmark case of Rex v. Bourne in Britain in 1938 helped to define “lawful” abortion when the court acquitted a doctor who performed an abortion on a rape victim to prevent the woman from becoming a “physical and mental wreck” [8]. This ruling established precedent for protection of doctors performing abortion, and was important to the establishment of the health exception. We were unable to find any cases in which a woman was unlawfully denied an abortion under the health exception in Britain. Numerous cases have been brought before Colombia’s Courts on behalf of women who are illegally refused an abortion or who are subject to unjustified delays, requests for unnecessary authorization, institutional conscientious objection, and other such barriers to care. Colombia’s Constitutional Courts have ruled in favor of these women [9]. This history of abortion jurisprudence was viewed by key informants as critical to defining, clarifying, and ultimately expanding the use of the health exception in Colombia. As one respondent explained,

“Following the [2006] sentence, there has been a significant volume of jurisprudence, tutelary sentences, constitutional sentences, and fundamental rights laws that have helped us in supporting the execution of that sentence” [*Provider, Colombia*].

In Mexico, most high-profile cases argued on behalf of women improperly denied legal abortion are for the rape exception, which is law in all of Mexico’s 32 states. We were only able to find one high-profile case argued under the health exception [27]. Women in Mexico are not only denied abortion under legal indications, but are also illegally targeted when abortion is suspected, such as stillbirth or miscarriage [11]. Jurisprudence affirming and clarifying a woman’s legal right to abortion under the health exception in Mexican states is lacking, and the absence of federal law makes it harder for courts to establish country-wide precedence.

## Discussion

The health exception is often pointed to as a means to expand access to legal abortion in restrictive settings [28]. However, our study reveals that the health exception is used broadly in Britain, somewhat in Colombia, and almost never in the 14 Mexican states with the health exception law. Our findings indicate that in addition to liberalizing abortion laws to include exceptions such as the health exception, interpretation and application of the law are pivotal to expanding access to safe abortion.

Our study aimed to identify the main differences and similarities affecting the use and interpretation of the health exception. We identified five overarching factors that affect varied application of the health exception: 1) comprehensiveness of the health exception law including the explicit mention of mental health, 2) funding of abortion within the public sector, 3) knowledge of and attitudes toward the health exception among the public and providers, as well as guidelines for provision of abortion, 4) dissemination of information about the health exception law, and 5) a history of court cases that protect women and clarify the law.

These findings are in line with other studies analyzing abortion access in different settings. Our findings support previous work on the importance of public funding for abortion to increase access [29]. Prior work has also highlighted the inclusion of mental health risk, jurisprudence and regulatory decisions regarding abortion law, and dissemination of information on the health exception as necessary for the expansion of abortion access in Latin America [28]. Analysis of the recent United States Supreme Court ruling regarding the unconstitutionality of Texas abortion restrictions acknowledges the importance of court cases in establishing precedent for abortion access [30], and a number of studies have focused on physician and public knowledge of abortion law as key to expansion [31, 32]. Our research builds upon this foundation to further analyze heterogeneous application of the health exception law and elucidate strategies for full implementation.

Expanding access to legal abortion under the health exception requires multi-faceted advocacy and policies. Advocates from many countries, including Britain, have argued for the removal of abortion law from the criminal code entirely, calling into question whether legalization is a necessary pre-cursor to expanded access [33]. Where abortion is legal under the health exception, following are our recommendations to expand access to abortion, as organized by theme:

1. *Use and Comprehensiveness of the Health Exception*
  Laws should be revised to explicitly mention mental health, in accordance with the WHO definition of health
  Experiences from countries where the health exception is broadly and legitimately applied (i.e. Britain) should be disseminated and set as a model for health systems where the law is underutilized or where newly liberalized abortion laws are being implemented
2. *Public Sector*
  Public health systems should be strengthened to expand care to vulnerable populations
  Public health systems should position the right to abortion within the human right to health more broadly, and work toward progressive realization of both
3. *Knowledge of and Attitudes toward the Health Exception Law*
  Barriers to information among women and providers should be mitigated. This may include information campaigns, comprehensive guidelines, and abortion education in medical schools
  Strong referral systems should be in place for providers who object to abortion provision
4. *Dissemination of Information*
  Ministries of Health should administer clear and comprehensive guidelines to providers about provision of abortion under the health exception
5. *Jurisprudence*
  Illegal refusals of legal abortion under the health exception should be documented by partners on the ground, and advocates should litigate on behalf of women who have been denied access to care under the health exception

This study has limitations. As an observational study, we cannot infer causality between identified factors and use of the health exception law in the three settings. Secondly, we recruited a small sample of key informants via convenience sampling that over-represent providers from urban settings with liberal attitudes toward abortion law. We aimed to adjust for this selection bias in our interview guide by asking questions about general attitudes, use, and interpretation of the health exception, and we believe providers know well the contexts in which they work. Our quantitative data is also limited by poor reporting of abortion in Mexico and Colombia; however, the statistics presented here do accurately reflect official abortion reporting in the three countries and can be appropriately used to reflect overall patterns of frequent or infrequent use of the health exception law. Finally, the results of this study are limited to the three countries under analysis: Britain, Colombia, and Mexican states. Our conclusions may not extend to other countries with similar health exception laws, such as members of the UK Commonwealth. A strength of our study is the use of diverse data sources which allowed us to triangulate information and verify findings.

It is important to consider potential challenges to the current legal framework, which could occur when attention is brought to further liberalization of abortion. However, full implementation of existing law, such as the health exception, is a key way to expand legal access to abortion. The recommendations outlined above can help to prevent challenges to the current legal status of abortion and encourage full implementation of the law. While not all recommendations will be feasible in all settings, nor swift, we believe the range of themes that emerged in our analysis allow other countries with similar laws to incorporate these lessons learned. In some settings, our best efforts may focus on only one of these facets, or, where multiple factors are addressed, will need to be approached individually and at different times.

## Conclusions

Application of the health exception in Britain is comprehensive, with clear and inclusive language, widespread dissemination, and broadly accessible information. Colombia, while thorough in their dissemination of the law, rigorous guidelines, and human rights-based approach to abortion law, is lacking in its explicit mention of mental health, and the improper denial of legal abortion to many women in the country. Mexican states with the health exception suffer these same limitations, as well as poor dissemination and a lack of guidelines.

As advocates in Colombia, Mexico, and Britain attest, the health exception is a valuable tool for expanding legal access to abortion. However, many women live in countries where abortion is legal but access is unrealizable. As our paper evinces, the interpretation of the law and the implementation of services – in addition to liberalizing abortion law – are pivotal to expanding access to legal abortion. We must incorporate lessons from countries where the health exception is broadly applied to ensure real access to abortion.

## Abbreviations

IMSS: Instituto Mexicano del Seguro Social
INEGI: Instituto Nacional de Estadística y Geografía
IRB: Institutional Review Board
ISSSTE: Instituto de Seguridad y Servicios Sociales de los Trabajadores del Estado
NGO: Non-governmental organization
NHS: National Health Service
RIPS: Sistema de Información de Prestaciones de Salud
SSA: Secretaria de Salud
WHO: World Health Organization
